# Multi-layer graphene oxide synergistically modified by two coupling agents and its application in reinforced natural rubber composites

**DOI:** 10.1039/c8ra05016c

**Published:** 2018-08-23

**Authors:** Meng Jiang, Yuzhu Xiong, Bai Xue, Qingpo Zhang, Qian Wan, Hailong Zhao

**Affiliations:** College of Materials and Metallurgy, Guizhou University Guiyang 550025 China xyzhu789@126.com; Guizhou Province Engineering Laboratory for Rubber Composites, Guizhou University Guiyang 550025 China bxue@gzu.edu.cn

## Abstract

Multi-layer graphene oxide (MGO) was co-modified with bis-(*P*,*P*-bis-ethylhexyldiphosphato)-ethanediolato titanate triethanolamino chelate solution (NDZ-311w) and bis-(γ-triethoxysilylpropyl)-tetrasulfide (Si-69). Then the co-modified MGO was incorporated into natural rubber (NR) by conventional two-roll mill mixing to prepare MGO/NR composites. The large macromolecule of NDZ-311w is able to efficiently intercalate the layers and increase the interlamellar space of MGO, subsequently resulting in the exfoliation of MGO into thinner sheets with better dispersity. Moreover, the oxygen-containing polar groups of MGO can be largely consumed by Si-69, which enhances the interfacial interaction between MGO and the NR matrix and improves the mechanical properties of the MGO/NR composites. Compared to pure natural rubber, the tensile strength, the stress at 300% strain, and tear resistance of co-modified MGO/NR composites are increased by 26%, 98% and 15%, respectively.

## Abstract 2

Multi-layer graphene oxide synergistically modified by two coupling agents can effectively reinforce natural rubber composites.

## Graphical abstract

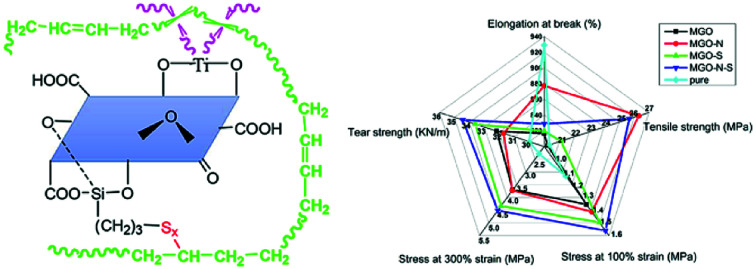

## 1. Introduction

Natural rubber (NR), as an important biopolymer, has excellent chemical and physical properties, for example, high elasticity at room temperature. However, NR usually needs to be reinforced with enhancing fillers to improve its shortcomings, such as poor strength, low modulus, and poor wear resistance. Graphene oxide (GO) is one of the new two-dimensional reinforcing and functional fillers for rubber nanocomposites, due to ultra-high mechanical strength, large surface area, low density, and high thermal conductivity.^1–4^ The performances of GO/NR composites can be influenced by many factors. The dispersion of GO and the compatibility of GO with a natural rubber matrix are the two important research directions for the preparation of GO/NR nanocomposites.

GO/NR nanocomposites have gained wide attention. Many scholars have used various kinds of surfactants or coupling agents to improve the dispersion of GO^5–9^ and the interface interaction between GO and the NR matrix.^10–14^ Ma *et al.*^15^ modified GO with a silane coupling agent by solution blending, which improved the dispersion of GO in silicone rubber. The results showed that the mechanical properties and thermal properties of GO/silicone rubber composites were greatly improved. Zhan *et al.*^16^ prepared natural rubber/graphene (NR/GE) composites by an ultrasonically-assisted latex mixing and *in situ* reduction process. GO was dispersed in natural rubber latex using an ultrasonic field and then *in situ* reduced, followed by latex coagulation to obtain the NR/GE masterbatch. This process produced much better dispersion and exfoliation of GE in the matrix and contributed to an increase in the tensile strength. Compared with pure rubber, the tensile strength and tear strength for NR/GE composites were increased by 47% and 50%, respectively. Li *et al.*^17^ incorporated GO that was modified with two differently terminated silane coupling agents into an epoxy resin to prepare nanocomposites. The results showed that the Young's modulus and tensile strength of amino-functionalized GO/epoxy (APTS-GO/epoxy) composites were greatly improved, and the fracture toughness and fracture energy of epoxy-functionalized GO/epoxy (GPTS-GO/epoxy) composites were nearly doubled at 0.2 wt% epoxy-functionalized GO loading.

In this paper, multilayer graphene oxide (MGO) was co-modified by NDZ-311w and Si-69, which takes advantage of the two coupling agents to create a synergistic modification method. The oxygen-containing functional groups of MGO are depleted by the coupling agents, and the hydrophobicity is largely enhanced. MGO synergistically modified by NDZ-311w and Si-69 can be more effectively stripped and the compatibility between the co-modified MGO and NR matrix is largely improved. Then, the MGO/NR masterbatch was fabricated by mixing the modified MGO aqueous dispersion with NR latex, followed by coagulation. After that, MGO/NR composites were obtained by introducing MGO/NR masterbatch into NR matrix and then vulcanization. The test results indicate that the tensile strength, modulus at 300% strain and tear resistance of co-modified MGO/NR composites are greatly improved by the introduction of MGO.

## 2. Experimental

### 2.1 Materials

Natural rubber (NR) was purchased from Yunnan Natural Rubber Industry Co. Ltd. (Kunming, China). Natural rubber latex (NRL, solid content is 62 wt%) was obtained from Shanghai Nessen international trading Co. Ltd. (Shanghai, China). Multi-layer graphene oxide (MGO) was bought from Suzhou Tanfeng Graphene technology Co. Ltd. (Suzhou, China). Bis-(*P*,*P*-bis-ethylhexyldiphosphato)-ethanediolato titanate triethanolamino chelate solution (NDZ-311w) was supplied by Nanjin Chuanshi Chem. Co. Ltd. (Nanjin, China). Bis-(γ-triethoxysilylpropyl)-tetrasulfide (Si-69) was purchased from the Green Wei Plastic Products Co. Ltd.(Dongguan, China). Calcium chloride (CaCl_2_), anhydrous ethanol, glacial acetic acid were provided by Chongqing Chuandong Chemical Co. Ltd. (Chongqing, China). Other commercially available reagents including zinc oxide (ZnO), stearic acid (SA), antioxidant *N*-isopropyl-*N*′-phenyl-4-phenylenediamin (4010NA), accelerator tetramethylthiuramdisulfide (TMTD), diphenylhydrazine (D), 2-mercaptobenzothiazole (M) accelerator 2,2′-dibenzothiazoledisulfde (DM) and sulphur were used without purification.

### 2.2 Preparation of modified MGO

Firstly, the ethanol solutions of coupling agents with a 5 wt% concentration were prepared at room temperature, and the pH value of the solutions was adjusted to 5.0 by using glacial acetic acid. Then, a certain amount of MGO was added to the 90 vol% ethanol solution to prepare the MGO suspension through ultrasonic treatment for 45 min. Afterwards, the ethanol solution of coupling agent was added into the MGO suspension, followed by magnetic stirring at 60 °C for 4 h. Finally, the solid content was separated from ethanol solution by vacuum filtration and then washed with ethanol solution at least three times to remove excess reactants. The modified MGO powder was obtained by drying in a vacuum oven at 80 °C for 12 h. MGO–N, MGO–S and MGO–N–S were modified with NDZ-311w, Si-69, and both coupling agents, respectively.

### 2.3 Preparation of modified MGO/NR nanocomposites

The modified MGO powder was dispersed in water by ultrasonic treatment to produce the modified MGO suspension and then a certain amount of the aforementioned suspension was dropped in NR latex with stirring for 1 h, followed by the coagulation with adding 2 wt% CaCl_2_ solution. The coagulation isolated by filtration was further dried under oven at 60 °C for the constant weight. Subsequently, the masterbatch, NR matrix and all other agents were mixed by an open two-roll mill at room temperature, according to Table 1. As control experiments, unmodified MGO/NR and pure NRL/NR composites were prepared by the same process. All the samples were cured at 143 °C up to their optimum cure time (*t*_90_) with 10 MPa, and the vulcanized samples were stored at room temperature for at least 24 h before testing.

**Table 1:** Curing formula of MGO/NR nanocomposites^a^

Ingredient	phr
Natural rubber (NR)	75
Natural rubber later (NRL)	25
MGO (modified or unmodified)	3
Zinc oxide (ZnO)	5
Stearic acid (SA)	4
Antioxidant *N*-isopropyl-*N*′-phenyl-4-phenylenediamin (4010NA)	1.5
Accelerator tetramethylthiuramdisulfide (TMTD)	0.32
Diphenylhydrazine (D)	0.5
2-Mercaptobenzothiazole (M)	2.21
Accelerator 2,2′-dibenzothiazoledisulfde (DM)	1.96
Sulphur	1.71

a Phr, parts per hundred of natural rubber by weight.

### 2.4 Characterizations

Fourier transform infrared spectrometer (FTIR, Nicolet 6700, Thermo Scientific Co. Ltd., USA) was used to analyze the functional groups of the modified MGO. X-ray photoelectron spectrometer (XPS, K-Alpha, Thermo Scientific Co. Ltd., USA) was applied to analyze the chemical elements of the modified MGO. The crystalline structure of the modified MGO was analyzed by a X-ray diffractometer (XRD, PANalytical B.V Co. Ltd., Netherlands) with Cu Kα radiation under a voltage of 40 kV and a current of 30 mA. Atomic force microscope (AFM) images was taken by a Nanoscope III D Multimode scanning probe microscope (Dimension ICON, Bruker Co. Ltd., USA) in a tapping mode. The modified MGO dispersion was coated onto a freshly exfoliated mica substrate and dried at room temperature to prepare the testing samples. Dynamic mechanical performance analysis was carried out on a rubber process analyzer (RPA 2000, Alpha Technologies, USA) with mixed rubber. All tests were conducted by using a strain sweep test with monitoring strain from 0.7% to 400% at 1 Hz frequency with 60 °C. A universal testing machine (Hegewald & Peschke Co. Ltd., Germany) was used to measure the tensile and tear properties of the MGO/NR composites at a uniform crosshead speed of 500 mm min^−1^ according to on GB/T 528-1998 (tensile property) and GB/T 529-1999 (tear resistance), respectively. The reported values including tensile strength, elongation, stress at 300% strain, stress at 100% strain and tear strength were recorded as the averages of five tests. The morphologies of modified MGO and MGO/NR composites samples were observed using a JSM-7500F scanning electron microscope (SEM) (JEOL Ltd., Japan).

## 3. Results and discussion

### 3.1 Characterizations of modified MGO

**Fig. 1:**
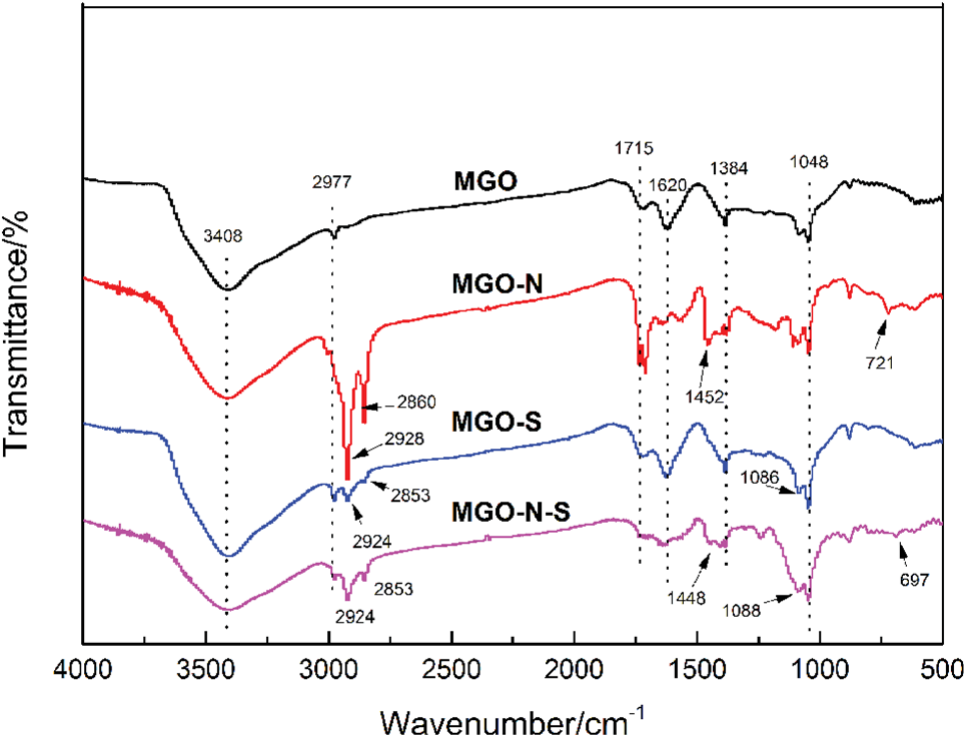
FTIR spectra of unmodified and modified MGO.

To shed light on the interaction between MGO and coupling agents, FTIR spectra were used to detect the functional groups of the modified MGO, which is shown in Fig. 1. In the spectrum of MGO, the broad peak at 3408 cm^−1^ results from the stretching vibration of –OH, while sharp peaks at 2977 cm^−1^, 1715 cm^−1^, 1620 cm^−1^, 1384 cm^−1^ and 1048 cm^−1^ can be attributed to the stretching vibration of –OH in the –COOH, stretching vibration of C=O, stretching vibration of C=C, O–H deformation vibration, and stretching vibration of C–O from the C–O–C,^18^ respectively. For MGO–N, besides the above-mentioned characteristic peaks of MGO, obvious peaks at 2928 cm^−1^, 2860 cm^−1^, 1452 cm^−1^ and 721 cm^−1^ related to the stretching vibration of –CH_3_, the stretching vibration of –CH_2_, the asymmetric bending vibration of –CH_3_ and the characteristic peaks Ti–O,^19,20^ respectively, are observed, which indicates that NDZ-311w are successfully bounded on MGO surface. For MGO–S, the new sharp peak observed at 1086 cm^−1^ is related to the stretching vibration of Si–O,^21,22^ which illustrates that Si-69 was also successfully bounded on MGO–S surface. The spectrum of MGO–N–S is a combination of MGO–N and MGO–S spectra. In addition, the stretching vibration of –OH at 3408 cm^−1^ has been reduced, which demonstrates that the hydrophobicity of MGO–N–S is greatly improved.

**Fig. 2:**
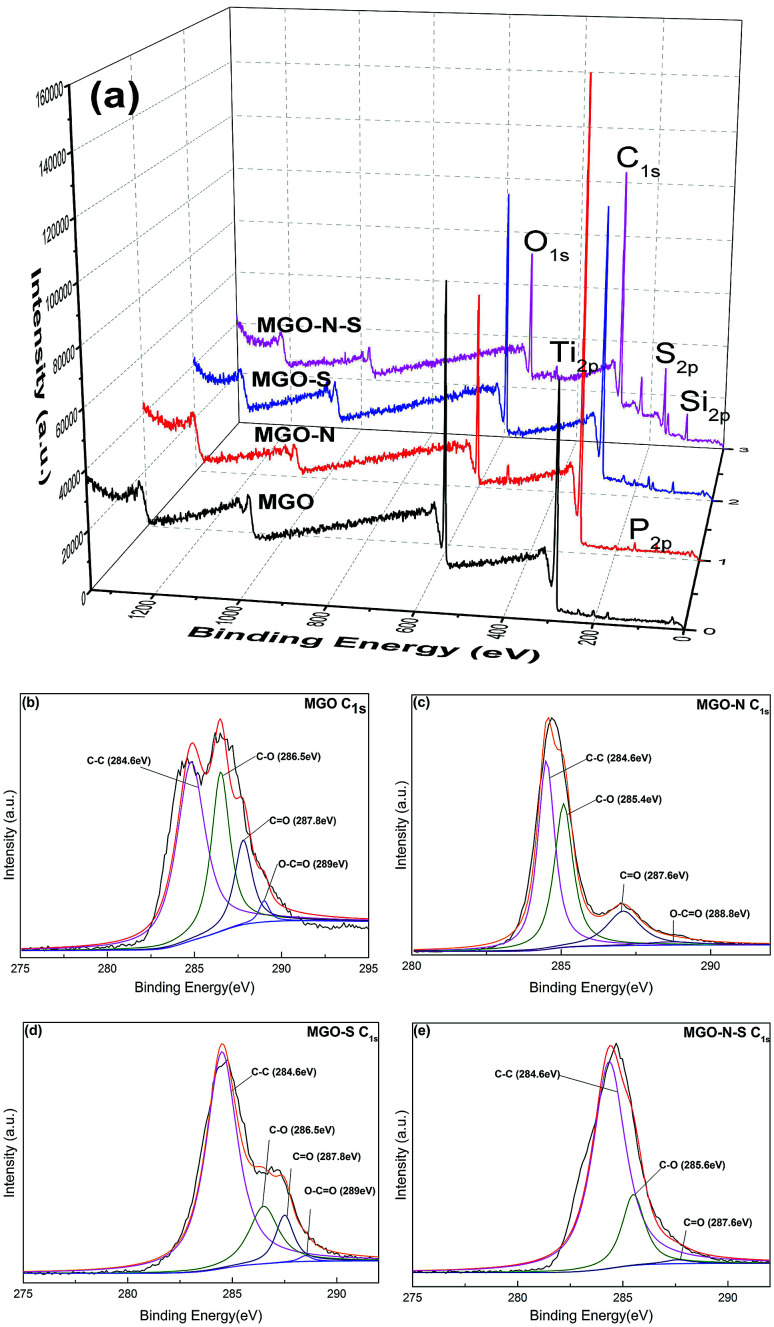
XPS spectra of MGO (a) and C_1s_ peak of MGO ((b) MGO, (c) MGO–N, (d) MGO–S, (e) MGO–N–S).

In order to further understand the interactions between coupling agents and MGO, XPS has been used to detect the surface chemical changes of MGO. Fig. 2 shows XPS survey spectra of the MGO samples. As shown in Fig. 2(a), Only C_1s_ (284.8 eV) and O_1s_ (532.5 eV) peaks are seen in the XPS survey spectrum of MGO,^23^ while P_2p_ (134.8 eV) and Ti_2p_ (459.7 eV) peaks are clearly observed in the XPS survey spectrum of MGO–N,^24,25^ Si_2p_ (101.7 eV) and S_2p_ (163.5 eV) peaks appear in the XPS survey spectrum of MGO–S,^26^ and all the mentioned-above peaks can be observed in the XPS survey spectrum of MGO–N–S. This phenomenon illustrates that MGO are successfully modified by NDZ-311w and Si-69. Moreover, the O/C ratio for MGO is 0.38, while the O/C ratio of MGO–N–S decreases to 0.24. The significant decrease in the oxygen content suggests that oxygen-containing functional groups are largely consumed by NDZ-311w and Si-69.

The XPS C_1s_ peaks were fitted by a multipeak Lorentzian fitting program (XPS peak) which are shown in Fig. 2 (b), (c), (d), and (e), respectively. The C_1s_ core level spectra of MGO shows all the peaks at C–C (284.6 eV), C–O (286.5 eV), C=O (287.8 eV) and O–C=O (289.0 eV).^27,28^ Although the C_1s_ peaks of MGO–N, MGO–S, and MGO–N–S exhibit the same oxygen functionalities (C–O (286.5 eV), C=O (287.8 eV) and O–C=O (289.0 eV)), their peak intensities are much smaller than those of MGO (especially, the C_1s_ peak in O–C=O (289.0 eV) of MGO–N–S cannot be detected), due to the effective reaction of MGO with NDZ-311w or/and Si-69. In addition, the C_1s_ peaks at the C–O (286.5 eV) and C=O (287.8 eV) shift to lower binding energy 285.4 eV and 287.6 eV for MGO–N, owing to the long alkyl group of NDZ-311w, implying the increase of electron density. These XPS results further demonstrate that MGO is successfully functionalized by the two coupling agents, which is in agreement with FTIR results. The drastically decreasing oxygen-containing functional groups of MGO lead to the improve hydrophobicity and interface interaction with NR matrix.

**Fig. 3:**
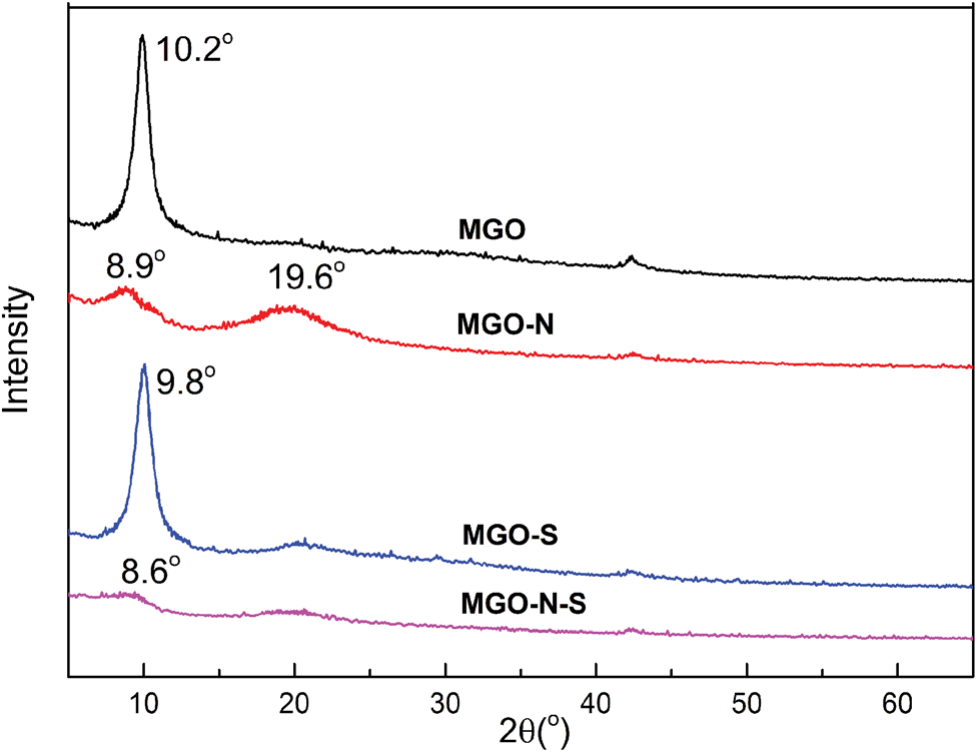
XRD patterns of unmodified and modified MGO.

The XRD patterns of the MGO, MGO–N, MGO–S and MGO–N–S are shown in Fig. 3. The interlayer distance can be calculated from the XRD data and hence the exfoliation of the MGO is assessed. The sharp diffraction peak around 10.2° for MGO shows the basal spacing of 0.87 nm. The MGO sheet is difficult to disperse because of the strong van der Waals force and electrostatic force between the sheets of graphite.^29,30^ The MGO–N shows a weak and broad diffraction peak at 2*θ* = 8.9°, assigned to the basal spacing of 0.99 nm. This phenomenon illustrates that the huge molecules of NDZ-311w destroy the crystal structure of MGO and insert the MGO interlayer. An additional peak at 19.6° suggests that the MGO cannot be fully exfoliated by NDZ-311w.^31,32^ The sharp diffraction peak around 9.8° for MGO–S shows that the basal spacing of MGO was 0.90 nm, which illustrates that Si-69 can not effectively exfoliate MGO. MGO–N–S does not shown any obvious diffraction peak, indicating that MGO–N–S is in an exfoliated state due to the synergistic modification of NDZ-311w and Si-69.^33,34^

**Fig. 4:**
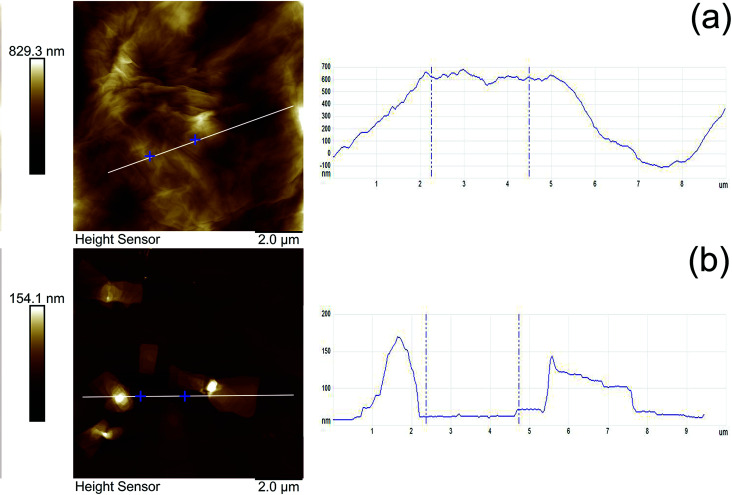
AFM images and the corresponding height profiles of (a) MGO and (b) MGO–N–S.

The morphology of MGO and MGO–N–S has been analyzed by AFM. Fig. 4 presents the tapping-mode AFM photos and the corresponding height profiles. The results show that the average thickness of MGO and MGO–N–S is 370 nm (Fig. 4(a)) and 54 nm (Fig. 4(b)), respectively, which indicates that MGO can be exfoliated into thinner GO sheets by the synergistic modification of NDZ-311w and Si-69. Moreover, the size of MGO is largely decreased from more than 10 μm to about 4 μm. In combination with the FTIR, XPS and XRD results, this phenomenon can be explained by that large macromolecule of NDZ-311w is able to efficiently intercalate the layers and increase the interlamellar space of MGO, and Si-69 can consume the oxygen-containing functional groups of MGO and reduce the interaction between MGO lamellas. In consequence, the exfoliation of MGO into relatively thin GO sheets is achieved as expected.

**Fig. 5:**
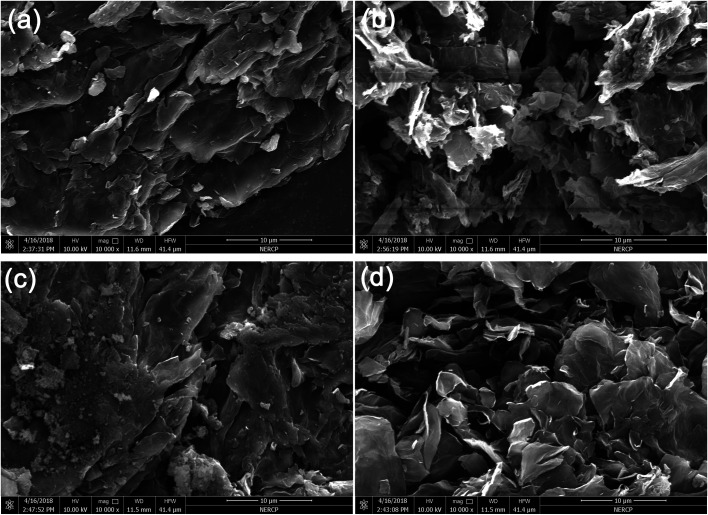
SEM images of MGO sheets (a) unmodified MGO, (b) MGO–N, (c) MGO–S, (d) MGO–N–S.

SEM has been applied to assistantly characterize the morphology of MGO and modified MGO. As shown in Fig. 5(a), MGO sheets are accumulated as a chunk due to the strong interaction between MGO sheets, and hence, the dispersity of MGO is relatively poor. The morphology of MGO–N, MGO–S and MGO–N–S are shown in Fig. 5(b–d), respectively. Obviously, the MGO is exfoliated into thinner and smaller GO sheets, which is consistent with the results of AFM.

**Fig. 6:**
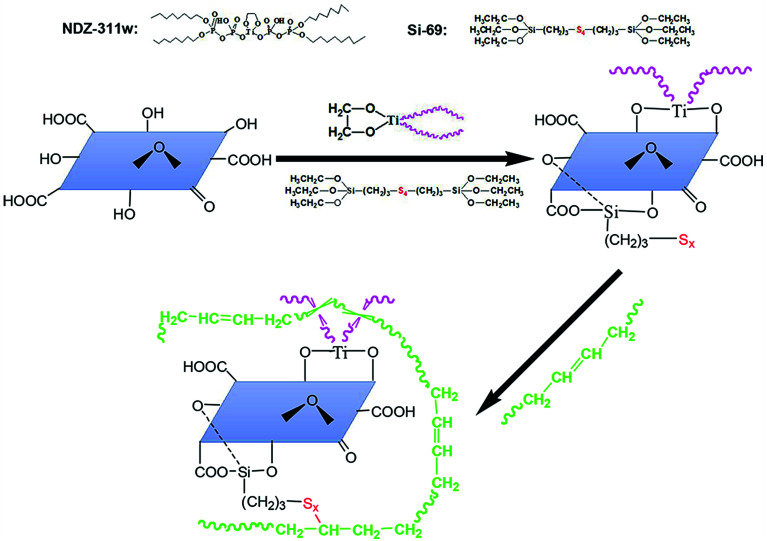
Schematic of proposed synergetic modification mechanism of MGO.

All these above results confirm that we have obtained an expected exfoliated dispersion structure of MGO. The reinforcing efficiency of MGO in composites depends on not only the dispersion of the MGO sheets in the matrix but also the interface interaction between the MGO sheets and the matrix. Considering that the majority of the functional groups of MGO are carbonyl and carboxyl groups at the sheet edges and the sulfide groups of Si-69 can be reacted with NR molecules, the Si-69 is introduced. And organic modifier NDZ-311w is also introduced because its huge molecule can insert into the interlayer of MGO and entangle with NR molecular chains. The interface bridges are thus built between the MGO sheets and the NR matrix by chemical bonding and physical entanglement points, as illustrated in Fig. 6.

**Fig. 7:**
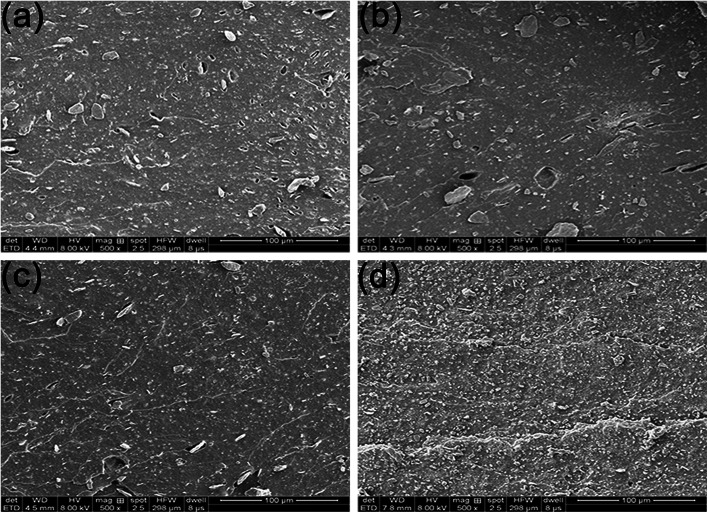
SEM images of tensile fracture surfaces of vulcanized modified-MGO/NR composites, (a) MGO/NR, (b) MGO–N/NR, (c) MGO–S/NR, (d) MGO–N–S/NR.

To reveal the reinforcing mechanisms, the morphology of the tensile fracture surface of vulcanized unmodified- and modified-MGO/NR composites was investigated by SEM. As shown in Fig. 7(a), MGO is aggregated in NR matrix and extracted from NR matrix, which states that the dispersity of MGO and the interface interaction between MGO and NR matrix are rather poor. As shown in Fig. 7(b), the thickness of MGO stacks is reduced due to the exfoliation of MGO into small MGO sheets after the modification by NDZ-311w. However, NDZ-311w lacks chemical crosslinking points to react with NR molecular chains which can provide powerful interface interaction between MGO and NR matrix. As shown in Fig. 7(c), there are some stacks on the tensile fracture surface, but there is no obvious MGO pulled out of the tensile fracture surface. The reason is that MGO can not be effectively exfoliated into few-sheet by using Si-69, but Si-69 can provide chemical crosslinking points to react with NR molecular chains. As shown in Fig. 7(d), there is no stacked texture and obvious extracted MGO on the tensile fracture surface. This appearance suggests that the synergistic modification by NDZ-311w and Si-69 improves the dispersity of MGO and enhances the interface interaction between MGO and NR matrix. All the results indicate that NDZ-311w and Si-69 co-modification MGO can achieve perfect exfoliation and dispersion in MGO–N–S/NR composites, which is consistent with the results of XRD and AFM analyses.

**Fig. 8:**
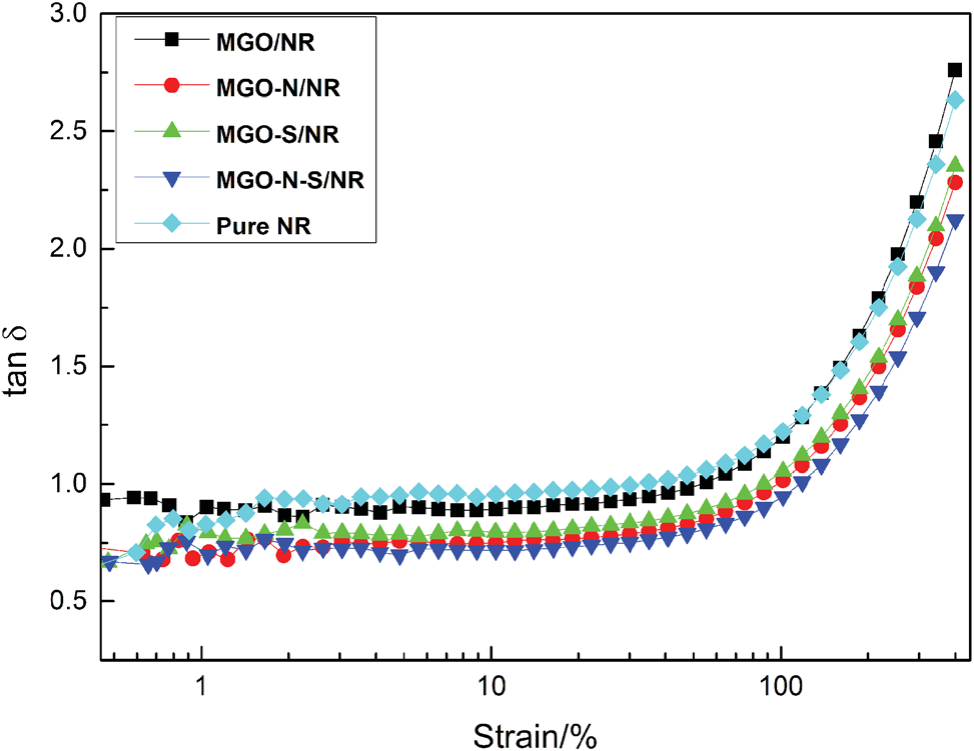
Loss factor *versus* strain curves of uncured MGO/NR composites and pure NR.

Fig. 8 shows the strain amplitude dependence of loss factor (tan *δ*) of MGO/NR, MGO–N/NR, MGO–S/NR, MGO–N–S/NR composites and pure NR. The dynamic behaviors of the composites show nonlinear rise of tan *δ* with the increasing strain amplitude. The interlayer of MGO can be inserted by NDZ-311w molecules, which can improve the dispersion of MGO. And the huge molecular structures of NDZ-311w can entangle with the molecular chains of NR and restrict the mobility of the NR molecular chains, so the tan *δ* of MGO–N/NR composites is slightly decreased, compared with MGO/NR composites and pure NR. Si-69 can improve the dispersion of MGO and provide some crosslinking points which will seriously restrict the mobility of the NR molecular chain. Thus the tan *δ* of MGO–S/NR is further decreased. The MGO–N–S/NR composites show the minimal tan *δ*, due to the synergistic modification of MGO by NDZ-311w and Si-69. When MGO is co-modified by NDZ-311w and Si-69, more coupling agents can be bonded onto the surface of MGO, which can provide larger numbers of entanglement and crosslinking points to restrict the mobility of the NR molecular chains.

**Fig. 9:**
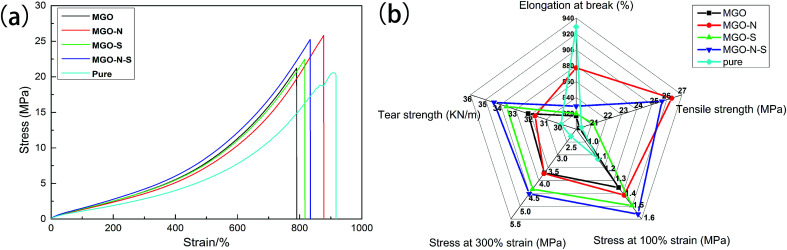
(a) The stress–strain curves of cured MGO/NR composites and pure NR, (b) elongation at break, tensile strength, stress at 100% strain, stress at 300% strain, and tear strength of MGO/NR composites and pure NR.

Fig. 9(a) displays the stress–strain curves of cured MGO/NR composites and pure NR. We notice that the tensile strength of MGO/NR composites is greatly increased due to a reinforcing effect of the MGO. However, there is a decrease in elongation at break of the composites, compared to pure NR material. MGO sheets well exfoliated by both NDZ-311w and Si-69 own pretty dispersion in NR matrix and enhanced interfacial interactions, which can cause the maximum stress transfer and the highest tensile strength. Mechanical properties including elongation at break, tensile strength, stress at 100% strain, stress at 300% strain and tear strength of MGO/NR composites and pure NR material are shown in Fig. 9(b). NDZ-311w can improve the elongation at break and tensile strength of the composites, and Si-69 can improve the stress at 100% strain, stress at 300% strain and tear strength of the composites. It is worth noting that the combining effect of NDZ-311w and Si-69 is higher than that of either NDZ-311w or Si-69 alone. The tensile strength, the stress at 100% strain, the stress at 300% strain and tear strength of MGO–N–S increase by as much as 26%, 38%, 98%, and 15%, respectively, over those of pure NR.

The excellent reinforcement by MGO sheets is not only due to the high degree of MGO sheets exfoliation in the rubber matrix, which allows a large contact area between the MGO sheets and the NR matrix, but also related to the strong interfacial interaction between MGO and NR matrix. To sum up, the improved mechanical properties are correlated with MGO homogenous dispersion, interfacial adhesion between MGO and NR matrix as well as synergistic modification of MGO by NDZ-311w and Si-69.

## 4. Conclusions

In this study, the synergistic reinforcing effect of NDZ-311w and Si-69 can effectively exfoliate MGO sheets, improve the dispersity of MGO in the NR matrix, and enhance the interfacial interaction between MGO and NR matrix. The modified MGO/NR composites were prepared by the conventional two-roll mill mixing method. In the MGO–N–S/NR composites, the chemical bridge between MGO and the NR matrix is built through NDZ-311w and Si-69, and the interfacial strength of the composites is significantly improved. The fabricated MGO–N–S/NR composites exhibit significantly better mechanical properties than the pure matrix. The tensile strength, the stress at 300% strain and tear strength of MGO–N–S are increased by 26%, 98%, and 15%, respectively.

## Conflicts of interest

There are no conflicts of interest to declare.

## Supplementary Material
